# The influence of salt reduction with encapsulated oleoresins on the quality of mayonnaise, mustard, and ketchup

**DOI:** 10.3389/fnut.2024.1456319

**Published:** 2024-09-19

**Authors:** Carmo Serrano, Margarida Sapata, Diogo Castelo-Branco, Ana Tasso, António Marques, Cláudia Viegas, Diogo Figueira, Norton Komora

**Affiliations:** ^1^Instituto Nacional de Investigação Agrária e Veterinária (INIAV, I.P.), Oeiras, Portugal; ^2^LEAF|Linking Landscape, Environment, Agriculture and Food Research Unit, Associated Laboratory TERRA, Intituto Superior de Agronomia, Universidade de Lisboa, Lisbon, Portugal; ^3^Mendes Gonçalves SA, Golegã, Portugal; ^4^Interdisciplinary Centre of Marine and Environmental Research, Terminal de Cruzeiros do Porto de Leixões, University of Porto, Matosinhos, Portugal; ^5^Division of Aquaculture, Upgrading, and Bioprospecting, Portuguese Institute for the Sea and Atmosphere, Lisbon, Portugal; ^6^H&TRC—Health & Technology Research Center, ESTeSL — Escola Superior de Tecnologia da Saúde, Instituto Politécnico de Lisboa, Lisbon, Portugal

**Keywords:** salt reduction, sauces and condiments, encapsulated oleoresins, quality parameters, sensory analysis

## Abstract

**Introduction:**

Mayonnaise, mustard, and ketchup are table sauces enjoyed worldwide, adding flavour and texture to many dishes. However, these products often contain high sodium content, which contributes to health issues such as high blood pressure and cardiovascular disease. To address these concerns, reducing salt content in the sauces has become a significant goal for both manufacturers and consumers.

**Objectives:**

This study investigates the effects of three formulations of microencapsulated (ME) oleoresins (F1, F2, and F3), derived from aromatic plants and spices, on the mineral content, physical–chemical properties, colour, and sensory profiles of mayonnaise, mustard, and ketchup.

**Results:**

The addition of ME ingredients resulted in significant reductions in salt content across all sauces, with reductions up to 50% in mayonnaise, 45% in mustard, and 52% in ketchup, aligning with EU sodium guidelines and allowing for a “reduced Na/NaCl content” nutrition claim. Potassium levels in mustard and ketchup were sufficient to support health claims related to blood pressure maintenance, while chloride content was reduced in ME formulations, better aligning with dietary reference values. Physical–chemical analysis revealed that ME ingredients had minimal impact on parameters like pH, lipid oxidation, and viscosity, although significant differences were observed in specific areas, such as the consistency of ketchup and chloride content in mustard and ketchup. The use of inulin, as a carrier agent, helped maintain the sauces rheological properties. Mustard showed the most similarity to the control in terms of physical–chemical parameters. Colour analysis indicated minimal changes in mayonnaise, moderate changes in mustard, and significant differences in ketchup, particularly with the ME-F3 formulation, where the light-yellow ME ingredients had a pronounced effect on the darker sauce. Despite these differences, the sensory analysis demonstrated that the overall sensory profiles of the ME formulations were similar the like control for all sauces. Mayonnaise showed the closest resemblance, while mustard had slightly lower scores in flavour and saltiness. Ketchup followed the same trend as mayonnaise, with no significant sensory differences compared to the control.

**Conclusion:**

These findings suggest that ME ingredients can be effectively used in condiment reformulation to achieve significant salt reduction without compromising sensory qualities, while also supporting health-related claims. By incorporating ME-based salt reduction strategies and exploring low-sodium alternatives, consumers can continue to enjoy their favourite sauces while minimising sodium intake. Embracing these changes not only benefits personal health but also aligns with the industry’s commitment to offering more nutritious options.

## 1 Introduction

The use of table sauces dates back to various cultures and regions worldwide. The Global Market for condiments, seasonings, and sauces will reach USD 181.0 billion by 2025 (1). Emulsion-based sauces (e.g., mayonnaise) and ready-made vegetable seasonings (mustard and ketchup) are mixtures of ingredients designed to enhance food flavour. These sauces are staples on kitchen tables and are especially prevalent in fast food, with mayonnaise being the most common, followed by ketchup and mustard.

Several studies have shown that these products contain very high levels of salt (2, 3). Regular consumption of foods and sauces high in sodium chloride can increase the risk of developing high blood pressure, heart disease, and stroke. This measure has prompted several governments and health organizations to advocate for salt reduction strategies to improve public health (4).

The WHO aims to reduce the average population’s sodium intake by approximately 30% by 2025 (5). In the European Union, national salt reduction initiatives have set maximum salt content targets for 12 food products, including sauces, condiments, and spices (6). In addition to setting industry targets, other policies have been considered to support reformulation. These include introducing taxes on high-salt foods and implementing labelling and communication strategies. Aligning these strategies with the food industry can improve public health and ensure consumer welfare.

Reducing salt in food can be challenging, given salt’s crucial role in flavour, texture, and preservation. Consequently, the food industry must continuously innovate to maintain traditional tastes and textures while reducing salt content. Numerous studies have developed new technologies and strategies, such as adjusting processing operations, selecting quality ingredients and using additives to achieve a 30–50% reduction in salt intake without compromising the product’s qualitative, physicochemical characteristics and sensorial aspects.

Table salt provides a salty taste to foods, and reducing it can alter the flavour profile, often resulting in acidic or sweet effects. Therefore, it is essential to imitate or replace the properties of salt flavours with other aromas or flavours. Although complex, this approach can significantly impact sodium intake from processed products. Testing is necessary to select substitute ingredients that best match the desired taste profile.

One strategy involves modifying formulations using mineral compounds like lithium, potassium, or ammonium chloride. However, these compounds can produce undesirable flavours, such as sour, metallic, and bitter notes (7). Additionally, potassium chloride (KCl) can pose health risks for individuals with Type 1 diabetes, necessitating limited consumption of this type of salt (8).

Another approach is the addition of organic sodium salts, such as acetate, glutamate, or citrate, although their salinity is significantly lower than sodium chloride (9). Non-mineral compounds, including amino acids (arginine, lysine, choline chloride) and flavour enhancers (10), such as monosodium glutamate (MSG), yeast extracts, alapyridine, arginine-apyridine derivatives, aromatic plants, spices (11), and hydrolyzed vegetable proteins (HVP) (12), have also been applied to enhance the salty taste of foods.

Taste enhancers work by activating receptors in the mouth and throat, helping to compensate for salt reduction. They stimulate receptors linked to the umami taste, improving the overall balance of taste perception in foods. According to the European Union, it is safe to use these taste enhancers. However, MSG has garnered a controversial reputation due to potential adverse effects in individuals with a vitamin B6 deficiency (13).

A promising new salt reduction technique involves combining flavour compounds from various foods and recipes, which may become increasingly important in future strategies.

Healthier alternatives, such as natural flavours derived from oleoresins, are increasingly used as food ingredients due to their uniformity in flavour, and aroma, microbiological stability, and ease of storage and transport. The rising demand for salt reduction in foods has significantly increased the use of oleoresins. However, challenges such as colour changes, low water solubility, and susceptibility to oxidation necessitate encapsulation, where sensitive compounds are enclosed within another substance (carrier) and released under specific controlled conditions.

Spray drying is a common method for obtaining encapsulated oleoresin flavours and aromas in powder form, suitable for incorporation into various matrices. Using inulin as a carrier meets technological and regulatory requirements, as it is biodegradable and has been Generally Recognized As Safe (GRAS) in the USA since 2002 (14). Microencapsulated oleoresins (ME), derived from aromatic plants and spices (15), have been applied in various food and culinary products (16), including fish (17), and fish products (18, 19). These applications allow for a salt reduction of over 25%, masking undesirable flavour components while maintaining or improving the flavour profile, texture, stability, and shelf-life.

This study investigates the effects of three formulations of microencapsulated (ME) oleoresins (F1, F2, and F3), derived from aromatic plants and spices, on the mineral content, physical–chemical properties, colour, and sensory profiles of mayonnaise, mustard, and ketchup. To our knowledge, this is the first study to use oleoresin-based microencapsulated (ME) ingredients to achieve a 25–50% salt reduction in these sauces, specifically targeting products manufactured by Mendes Gonçalves SA, a Portuguese company. The goal is to replace salt with encapsulated oleoresins to enhance flavour while meeting the functional, technical, and quality requirements of the sauces, aligning with consumer needs and preferences.

## 2 Materials and methods

### 2.1 Sauce ingredients

The sauce ingredients were provided by Mendes Gonçalves’. The control and ME ingredients (F1, F1-A, F1-B, F2, and F3) low salt mayonnaise, mustard and ketchup sauce samples were formulated as shown in Table 1, and the preparation is described in sections 2.2.1–2.2.3. The final product was transferred into an airtight bottle and packaged. All sauce samples were stored at room temperature (T = 20.00°C ± 0.05) until physicochemical and sensory analysis were carried out.

**Table 1:** Sauces and condiments formulations based on 1 kg for each treatment (w/w %) with varying salt levels and ME.

Ingredient	Mayonnaise	Mustard	Ketchup
Water	58.54 ± 2.27	71.98 ± 1.48	34.29 ± 1.01
β carotene (A160a)	0.02 ± 0.00	–	–
Oil (vegetable)	26.37 ± 1.03	–	–
Tomato concentrate	–	–	28.92 ± 1.30
Sugar	2.74 ± 0.48	2.36 ± 0.16	23.01 ± 1.04
Vinegar	5.98 ± 0.74	10.72 ± 1.11	11.56 ± 1.01
Salt	0.35 ± 0.15	1.85 ± 0.20	1.52 ± 0.09
F1	0.35 ± 0.15	1.85 ± 0.20	1.52 ± 0.09
F1-A	0.17 ± 0.01	–	–
F1-B	0.35 ± 0.15	–	–
F2	0.35 ± 0.15	1.85 ± 0.20	1.52 ± 0.09
F3	0.35 ± 0.15	1.85 ± 0.20	1.52 ± 0.09
Gum	0.3 ± 0.11	0.10 ± 0.006	0.19 ± 0.04
Mustard	–	11.94 ± 0.99	–
Starch	5.5 ± 0.81	2.66 ± 0.30	2.90 ± 0.97
Egg	4.56 ± 0.37	–	–

The salt content added for formulations F1-A and F1-B was 0.53% (w/w).

### 2.2 Products preparation (recipes)

#### 2.2.1 Mayonnaise sauce preparation

The ingredients listed in Table 1 were used to prepare 1 kg of mayonnaise sauce for each of the three batches. In a bowl, the starch, sugar, salt, and ME (F1, F2, and F3) were mixed until a homogeneous mixture was obtained. Using a food processor (Bimby, Model TM31, Germany), the colouring agent was first added to the water, followed by the powders, and the mixture was homogenised for 2 min. The egg yolk was then added at low speed, followed by the oil, and mixed for 6 min until the emulsion system was established. Finally, the vinegar was added and mixed for a further 5 min.

#### 2.2.2 Mustard sauce preparation

For the preparation of 1 kg of mustard sauce samples for each of the three batches, the ingredients listed in Table 1 were used, and the process was divided into two steps. In the first step, the mustard base was prepared by combining the mustard seeds with water (47.29% w/w) in a bowl and soaking them for at least 2 h. After soaking, the mustard seeds were transferred to a food processor (Bimby, Model TM31, Germany), where vinegar was added, and the mixture was milled for 5 min. The mixture was then dissolved in hot water at 90°C for 10 min.

In the second step, the mustard sauce was prepared by adding water, starch, sugar, salt or ME (F1, F2, and F3), and gum to the mustard base and blending for 5 min. The final product was transferred to an airtight jar and packaged.

#### 2.2.3 Ketchup sauce preparation

The ingredients listed in Table 1 were used to prepare 1 kg of ketchup sauce for each of the three batches produced. In a container, the powders—sugar, salt or ME (F1, F2, and F3), gum, and starch—were mixed until well combined. In a food processor (Bimby, Model TM31, Germany), water was added to the tomato concentrate, mixed with the powders, and heated at 90°C for 15 min. Vinegar was then added, and the entire mixture was stirred for an additional 5 min to combine all the ingredients.

### 2.3 Physical, chemical, and sensory analysis

The sauces and condiments were analysed by physicochemical, textural and sensory analysis to evaluate the quality and consistency of the products and to monitor the production process to ensure that the products meet regulatory requirements and consumer expectations concerning taste, texture, and safety. Each physicochemical test was performed on control and ME ingredients (F1, F1-A, F1-B, F2, and F3) samples of low salt mayonnaise, mustard and ketchup sauces, after replacing the salt content of the control with 50% of each ME (mayonnaise, mustard, and ketchup) and 25% (mayonnaise), three bottles of 200 g for each batch these sauce products were analysed as replicates for each batch (Table 2).

**Table 2:** Physicochemical parameters of mayonnaise (M), mustard (Mu), ketchup (K), and control (Co), containing different added ingredients (F1, F2, and F3), respectively.

	pH	Acidity (%)	Density (Pa s)	Chloride (gKg^−^1)	TBA (mgKg^−1^)	Viscosity (cP)	Consistency (cm/30 s)	Brix
M-Co	3.84 ± 0.00^c^	0.72 ± 0.04^a^	1.00 ± 0.01^a,b^	1.06 ± 0.04^c^	0.02 ± 0.00^a^	12653.33 ± 100.66^c^	–	–
M-F1	3.82 ± 0.01^b^	0.76 ± 0.02^a^	0.99 ± 0.00^a^	0.36 ± 0.00^a^	0.02 ± 0.00^a^	14346.67 ± 54.55^a,b^	–	–
M-F2	3.79 ± 0.01^a^	0.78 ± 0.03^a^	1.00 ± 0.00^a^	0.38 ± 0.01^a^	0.02 ± 0.00^a^	14933.33 ± 612.32^a^	–	–
M-F3	3.83 ± 0.00^b,c^	0.73 ± 0.04^a^	1.00 ± 0.00^a^	0.36 ± 0.00^a^	0.02 ± 0.00^a^	15186.67 ± 794.31^a^	–	–
M-F1A	3.78 ± 0.00^a^	0.73 ± 0.08^a^	0.99 ± 0.00^b^	0.55 ± 0.03^b^	0.02 ± 0.00^a^	14213.13 ± 477.21^a,b^	–	–
M-F1B	3.78 ± 0.00^a^	0.75 ± 0.03^a^	0.99 ± 0.00^b^	0.51 ± 0.02^b^	0.02 ± 0.00^a^	13387.67 ± 371.66^b,c^	–	–
Mu-Co	3.78 ± 0.00^b^	1.31 ± 0.02^a^	1.07 ± 0.00^a^	3.64 ± 002^c^	–	18200.00 ± 0.00^a^	–	–
Mu-F1	3.80 ± 0.01^a^	1.31 ± 0.04^a^	1.24 ± 0.31^a^	1.87 ± 0.00^a^	–	18617.67 ± 321.46^a^	–	–
Mu-F2	3.82 ± 0.00^c^	1.27 ± 0.02^a^	1.06 ± 0.00^a^	1.98 ± 0.00^b^	–	17300.00 ± 476.97^a^	–	–
Mu-F3	3.80 ± 0.00^a^	1.32 ± 0.01^a^	1.06 ± 0.00^a^	1.86 ± 0.00^b^	–	17916.67 ± 354.73^a^	–	–
K-Co	3.71 ± 0.00^a^	1.61 ± 0.01^b^	1.17 ± 0.00^a^	3.13 ± 0.00d	–	–	5.52 ± 0.36d	36.40 ± 0.44^a^
K-F1	3.73 ± 0.00^a^	1.55 ± 0.02^a^	1.17 ± 0.00^a^	1.66 ± 0.01^b^	–	–	3.68 ± 0.11^a^	38.43 ± 0.32^c^
K-F2	3.72 ± 0.00^a^	1.60 ± 0.01^b^	1.16 ± 0.00^a,b^	1.64 ± 0.00^a^	–	–	4.45 ± 0.07^c^	37.73 ± 0.46^b,c^
K-F3	3.73 ± 0.00^a^	1.57 ± 0.01^a^	1.16 ± 0.00^b^	1.71 ± 0.00^c^	–	–	4.05 ± 0.07^b^	37.13 ± 0.15^a,b^

Data are presented as the mean ± standard error of three experiments. *Samples with the same letters are not significantly different at 5%.

#### 2.3.1 Minerals

The salt content was measured by determining the potassium (K) and sodium (Na) levels using flame atomic absorption spectrophotometry (Spectr AA 55B spectrophotometer, Varian, Palo Alto, CA, United States) with a deuterium background correction, following the method described by (20). The concentrations were calculated using linear calibration curve generate from absorbance measurements of, at least, five different concentrations of standard solutions (KNO_3_ and NaNO_3_, dissolved in 0.5 M HNO_3_).

The conversion of sodium to salt content was calculated based on the equivalence that 1 g of sodium is approximately equal to 2.5 g of salt (21).

#### 2.3.2 Chloride

The chloride ion content of the mayonnaise, mustard, and ketchup analysis was determined by direct titration of a portion of the sample with 0.5 M AgNO3, according to Mohr’s method (22), using a salt analyser (SALT-Matic 23, Crison, Spain).

#### 2.3.3 Acetic acidity content and pH

Acetic acid content of the mayonnaise, mustard, and ketchup was carried out using a pH titrator (Easy Plus Easy, Mettler Toledo, United States). The pH of the sauces was determined using a pH meter (HI 2211 pH/ORP, Hanna Instruments, Romania).

#### 2.3.4 Lipid oxidation

The oxidative state of mayonnaise samples was monitored using the 2-thiobarbituric acid (TBA) method by Abeyrathne et al. (23). Briefly, samples (5 g) were mixed with trichloroacetic acid (TCA) and ethylenediaminetetraacetic acid (EDTA), then shaken at 25°C for 2 min. The supernatant was filtered, centrifuged, and mixed with TBA solution. After incubating in boiling water for 40 min, the absorbance was measured at 530 nm. A standard curve was prepared using tetraethoxypropane (TEP) dilutions, and all determinations were performed in triplicate. The TBA index, expressed as mg of malondialdehyde (MDA) per 1,000 g sample, was calculated accordingly Equation 1 as follows:


Equation 1 $$TBA=\frac{72\times c}{m\times v}\;\left(30+\mathrm{mH}\right)$$

where *c*, is the concentration of MDA, expressed in μm, *v*, is the volume in mL, *H* is the sample humidity, in %, and *m*, is the mass of the test sample, in g. The result becomes the arithmetic mean of two parallel determinations, rounded to the nearest tenth.

#### 2.3.5 Refractive index

The measurement of the refractive index to determine the ° Brix in ketchup condiments was performed using a refractometer (HI 96801, Hanna Instruments, Romania), at 20°C, after a calibration with distilled water.

#### 2.3.6 Viscosity, density, and consistency

Viscosity was measured at 20°C using a rotary viscometer (Ametek Brookfield DV2T, United States) equipped with spindle 7 at 100 rpm and spindle 6 at 20 rpm for mayonnaise and mustard, respectively. Viscosity units are given in centipoise (cP). The density of the mayonnaises was determined by gravimetric method using an Erichsen pycnometer (50 mL, Mod. 209/IV, Germany) and units were expressed in pascal seconds (Pa-s). The Bostwick consistency of ketchup was assessed using a Bostwick consistometer (model LD-BC). The Bostwick consistometer measures sample flow in a graduated trough. A 50 mL compartment, separated by a spring-loaded gate, is filled and levelled. At 20°C, the gate is opened, and a stopwatch is started. After 30 s, the position of the sample is recorded with the consistency units expressed in centimetres per 30 s (cm/30s) (24).

#### 2.3.7 Colour

The sauces colourimetry analysis was performed by measuring the colour parameters L* (luminosity between 0 - black and 100 - white), a* (reddish-green), b* (yellowish-blue) using a colourimeter Chroma meter CR-5 Konica Minolta colourimeter (Konica Minolta, Japan), using an illuminate D 65 and a 2° observation angle. The colour difference degree between the mayonnaise, mustard, and ketchup, containing different added ingredients, and control, ΔE_ab_* values was performed according to Macdougall (25) by means of the formula: ΔE_ab_* = √(ΔL*^2^ + Δa*^2^ + Δb*^2^) where ΔL*, Δa* Δb* represent the difference between each parameter for the sauces and condiments, where each product without added ingredients was used as control. The ΔE_ab_* can be defined as the numerical comparison of a sample’s colour to the control according to Colour difference ΔE–A Survey (26).

#### 2.3.8 Sensorial analysis

Sensorial analysis was conducted in accordance with ISO 8586:2023 Sensory Analysis (27). A panel of untrained consumers in a blind test, comparing against a target, using a 9-point hedonic scale, where a score of 5 indicated “equal to target” and the scale ranged from “worst” to “best.” The attributes measured and their descriptors were as follows: overall evaluation, taste, smell, appearance, texture and saltiness. Each product was assessed by at least seven different subjects and after microbiological tests, carried on samples using the company’s internal protocols, and the results confirmed that the samples were within safe parameters, ensuring the safety of the tasters.

### 2.4 Statistics analysis

The statistics results were submitted to one-way ANOVA using multiple comparison tests (Tukey HSD) to identify differences between groups. Statistical analyses were tested at a 0.05 level of probability. The range, mean and relative standard deviation (RSD) of each parameter were calculated using the software, StatisticaTM 12.0 (28).

## 3 Results and discussion

### 3.1 Minerals analysis

**Figure 1:**
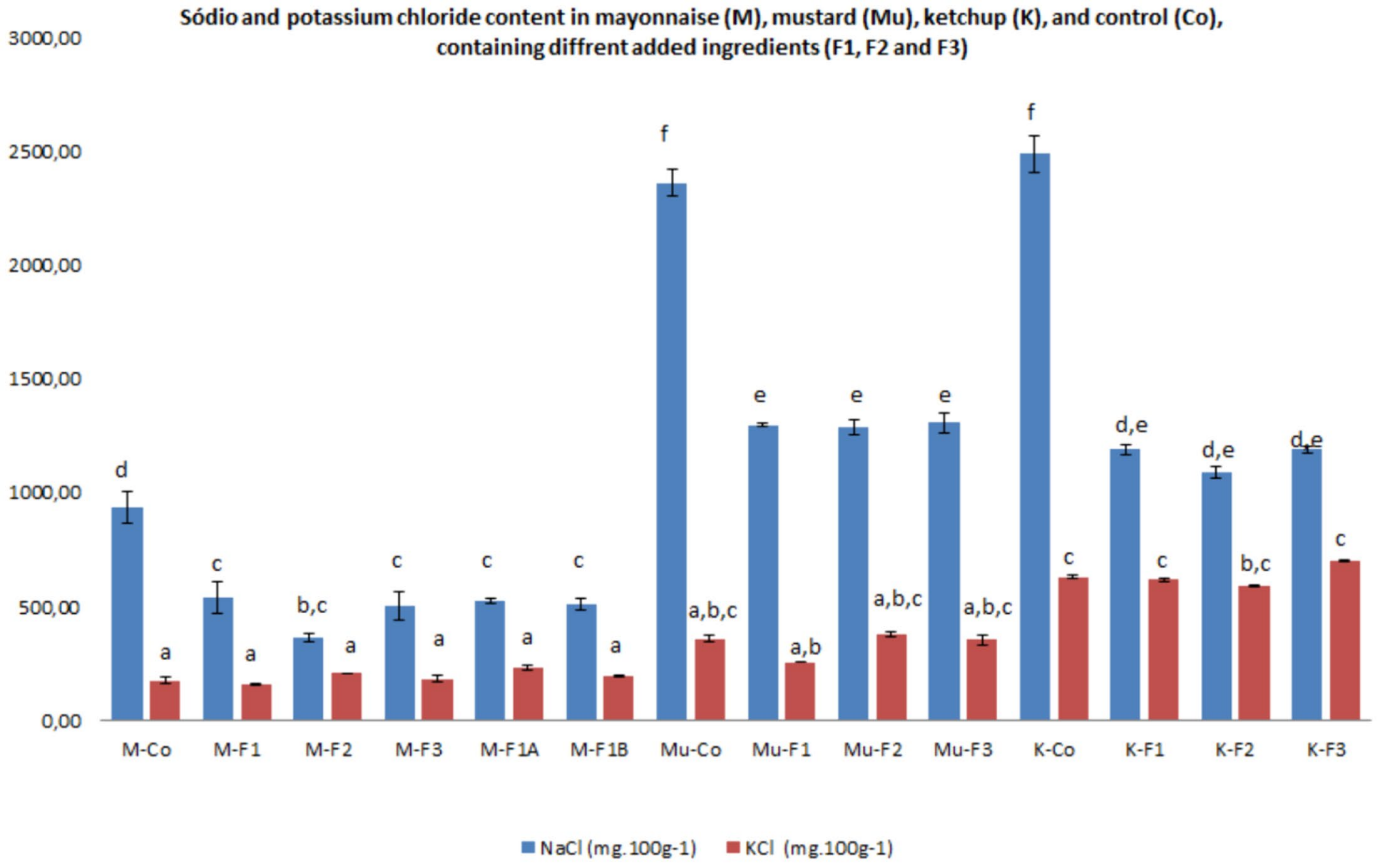
Sodium and potassium chloride content in mayonnaise (M), mustard (Mu), ketchup (K), and control (Co), containing different added ingredients (F1, F1A, F1B, F2, and F3), respectively. *Bars with the same letters are not significantly different at 5%.

The salt content (NaCl and KCl) in mayonnaise (M), mustard (Mu), and ketchup (K) (Figure 1) revealed significant differences (*p* < 0.05) with the addition of ingredients (F1, F2, and F3) compared to the control sauces (Co). This reduction in salt content was 50% for the various sauces and 25% in mayonnaise when two specific ingredients (F1-A and F1-B) were used.

For mayonnaises prepared with ME ingredients, the NaCl content ranged from 360 to 540 mg per 100 g, corresponding to 145–217 mg of sodium per 100 g. In contrast, the control mayonnaise had nearly double the salt content at 937 mg per 100 g, equivalent to 375 mg of sodium per 100 g. However, many commercially available mayonnaise sauces contain much higher sodium levels, averaging 603.6 ± 54.38 mg per 100 g, as reported by (29) in mayonnaise sauces without salt reduction. Among the ME ingredients, F2 resulted in the lowest total NaCl content, although no significant differences (*p* > 0.05) were observed when compared to other ingredients (F1, F1-A, F1-B, and F3). These results fall within the EU Pledge recommendation for all emulsion-based sauces, which sets a limit of 750 mg of sodium per 100 g. The reformulation of mayonnaise sauce using ME as NaCl replacement resulted in a significant reduction of 42–62% in sodium NaCl content. Consequently, the reduction in sodium content can be applied for the nutrition claim of “reduced Na/NaCl content” (reduction ≥25% compared to the control) can be applied to all formulations developed using ME (30), aligning with dietary recommendations and public health concerns related to high sodium intake, such as hypertension and cardiovascular diseases.

For mayonnaise the KCl content in the ME formulations ranged from 159.62 to 232.89 mg per 100 g, while the control averaged 172.72 mg per 100 g. The processing did not result in significant differences (*p* > 0.05) among the ingredients, indicating that KCl is not essential in these formulations. Additionally, the increased potassium content does not support a health claim related to maintaining normal blood pressure, as the sauces contain less potassium than required (30).

The control mustard sample contained 2,363 mg per 100 g, corresponding to 945 mg of sodium per 100 g, exceeding the recommended threshold by 26%. This sodium level is slightly lower than the 1,100 mg per 100 g found in other commercial mustard brands (31, 32), but nearly double the amount detected in the reformulated mustard with ME oleoresins. In contrast, mustard samples with ME ingredients had salts content ranging from 1,289 to 1,307 mg per 100 g, equivalent to 516–523 mg of sodium per 100 g, meeting the EU Pledge recommendation for emulsion-based sauces. No significant differences (*p* > 0.05) were observed among the different ME ingredients (F1, F2, and F3). The nutrition claim can also be applied to all ME formulations (30), as each resulted in approximately a 45% reduction in sodium content.

The KCl content in ME mustard formulations ranged from 256.11 to 378.30 mg per 100 g, while the control averaged 357.45 mg per 100 g. These values also showed no significant differences (*p* > 0.05) across the ingredient formulations, indicating that the addition of KCl to the F2 formulation is unnecessary. In this case, the potassium content in the mustard sauces (≥ 350 mg per 100 g) supports a health claim related to maintaining normal blood pressure, as the sauces contain the required potassium levels (30). This may be attributed to the potassium content in the mustard seasonings that can ranged between 693 and 774 mg per 100 g (33), used during sauce processing.

In ketchup, the salt content ranged from 1,088 to 1,191 mg per 100 g, corresponding to 435–477 mg of sodium per 100 g. As expected, the control sample presented almost double this amount, with 2,489 mg of salt per 100 g, corresponding to 996 mg of sodium per 100 g. This level of sodium is similar to that found in other brands of ketchup (2), but almost double the amount detected in mustard reformulated with any of the three ME oleoresin formulations. As with mustard, no significant differences (*p* > 0.05) were observed between the different formulation (F1, F2, and F3). Salt and sodium levels in samples containing oleoresins met the EU Pledge recommendation for emulsion-based sauces, while the control exceeded this limit by 33%. Therefore, using any of the ME formulations, a reduction of around 52% in sodium content is achieved, allowing the previous nutritional claim to be applied (30).

For ketchup, the KCl content in ME formulations ranged from 588.37 to 698.89 mg per 100 g, while the control sample averaged 620.96 mg per 100 g. No significant differences (*p* > 0.05) were observed among the different ingredient formulations, indicating that adding KCl to the F2 formulation is unnecessary. With potassium content exceeding 350 mg per 100 g, these sauces already support a health claim related to maintaining normal blood pressure, as they provide more than the required potassium (30). The elevated potassium levels may be attributed to the potassium content in the tomatoes, which may vary between 403.02 ± 254.41 mg per g (34) used during sauce processing.

The chloride content in the control sauces (1.1–3.7 mg per 100 g) was statistically higher than in the formulations developed with ME oleoresins (0.6–2.0 mg per 100 g). This suggests that these formulations better align with the Dietary Reference Values (DRVs) for chloride, which range from 1.7 to 3.1 g per day for children and adults, respectively (35).

### 3.2 Chemical and physical changes

Regarding the physical–chemical parameters (Table 2), no significant differences (*p* > 0.05) were observed between the control and the samples with ME ingredients in the pH of the ketchup, the lipid oxidation (TBA) of the mayonnaise, and the viscosity of the mustard. However, significant differences (*p* < 0.05) were found between the control and ME samples for the pH of the mustard, the chloride content of the mustard and ketchup, and the consistency of all three sauces.

For mayonnaise, there were no significant differences (*p* > 0.05) in the pH between samples F1-A and F1-B. Additionally, no differences (*p* > 0.05) were observed in acetic acidity among all samples. A low pH and consistent acetic acid levels indicate that reducing salt and using encapsulated oleoresins did not compromise the safety and preservation of the sauces. Since acetic acid is the predominant acid, these products are expected to be toxic and destructive to bacterial pathogens, particularly under conditions of low pH and high titratable acidity (36). Regarding density, no differences (*p* > 0.05) were found between samples F1, F2, and F3, as well as between samples F1-A and F1-B.

Concerning viscosity, no significant differences (*p* > 0.05) were observed between samples F2 and F3. Unlike findings reported by other authors (37), reducing the salt content and altering the type of salt did not have a significant effect (*p* > 0.05) on viscosity. This may be attributed to the inulin used as a carrier agent for encapsulating the oleoresins, which has gelling properties that helped maintain the rheological properties of the mayonnaise (38).

For mustard, no significant differences (*p* > 0.05) were found for all physic-chemical parameters of all samples, with this sauce being the one that most resembles the control.

For ketchup, no significant differences (*p* > 0.05) were found in acidity between samples F1 and F3, not in density between the control and the F1 sample. However, significant differences (*p* < 0.05) in ketchup consistency were found between the control and the formulations containing oleoresins ME. The ketchup with F1 oleoresins ME exhibited lower consistency but had higher total soluble solids compared to the control. Typically, higher consistency is expected to correlate with higher total soluble solids, not lower (39). This discrepancy may be due to the inulin used as a carrier agent in the oleoresins ME, which dissolves in water and increases the overall concentration of soluble materials in the product.

These results indicate that ME ingredients have a selective impact on the physical and chemical properties of these sauces.

### 3.3 Colour parameters

Table 3 shows the total colour difference (ΔE_ab_*) between samples with ME ingredients (F1, F1-A, F1-B, F2, and F3) compared to the control in the three types of sauces.

**Table 3:** Colour parameters (L*, a*, b*) and total colour difference (ΔE_ab_*) of mayonnaise (M), mustard (Mu), ketchup (K), and control (Co), containing different added ingredients (F1, F2, and F3), respectively.

	L*	a*	b*	ΔE_a,b_*
M-Co	90.00 ± 1.00^d^	3.00 ± 0.00^d^	22.67 ± 0.58^a,b,c^	–
M-F1	89.00 ± 1.00^d^	4.00 ± 0.00^d^	23.33 ± 0.58^b,c^	1.79 ± 0.42^a^
M-F2	89.67 ± 0.58^d^	4.00 ± 0.00^d^	21.67 ± 0.58^c^	1.53 ± 0.57^a^
M-F3	89.00 ± 1.00^d^	4.00 ± 0.00^d^	22.67 ± 0.58^a,b,c^	1.61 ± 0.65^a^
M-F1A	89.67 ± 0.58^d^	4.00 ± 0.00^d^	22.00 ± 0.00^a,b,c^	1.32 ± 0.21^a^
M-F1B	89.00 ± 0.00^d^	4.00 ± 0.00^d^	23.00 ± 0.00^b,c^	1.45 ± 0.00^a^
Mu-Co	69.33 ± 0.58^c^	2.00 ± 0.00^a^	49.67 ± 0.58^d^	–
Mu-F1	67.67 ± 0.58^c^	2.67 ± 0.58^a,b^	51.33 ± 0.58^e^	2.56 ± 0.38^a,b^
Mu-F2	69.00 ± 0.00^c^	2.00 ± 0.00^a,b^	51.67 ± 0.58^d^	2.27 ± 0.50^a,b^
Mu-F3	68.33 ± 0.58^c^	3.00 ± 0.00^a,b^	51.33 ± 0.58^d^	2.28 ± 0.25^a,b^
K-Co	22.00 ± 0.00^a,b^	25.67 ± 0.55^e^	20.33 ± 1.15^a^	–
K-F1	20.33 ± 0.58^a^	24.00 ± 0.00^c.d^	21.67 ± 0.58^a,b^	2.75 ± 0.60^b,c^
K-F2	22.00 ± 0.00^a,b^	25.00 ± 0.00^d,e^	23.67 ± 0.58^b,c^	3.40 ± 0.56^b,c^
K-F3	23.33 ± 1.53^c^	23.00 ± 1.73^c^	22.67 ± 2.08^a,b,c^	4.49 ± 0.98^c^

Data are presented as the mean ± standard error of three experiments. *Samples with the same letters are not significantly different at 5%.

For mayonnaise, the lowest ΔE_ab_* values were obtained, with no significant differences (*p* > 0.05) observed among all samples containing ME ingredients. Specifically, the ΔE_ab_* values ranging from 1 to 2 for mayonnaise with ME (F1-A and F2-B) and a 25% salt reduction, as well as for mayonnaise with ME (F1 and F2) and a 50% salt reduction, were unnoticeable except to experienced consumers (26).

The colour difference for mustard is noticeable within the range of 2 < ΔE_ab_* < 3.5. In contrast, for ketchup, the highest ΔE_ab_* value observed was 4.49 for ME-F3, indicating significant differences (*p* < 0.05) among the colour differences (3.5 < ΔEab* < 5) of all ketchup samples containing ME ingredients. This suggests that the light-yellow colour of the ME ingredients has a more pronounced impact on darker sauces like ketchup, resulting in higher ΔE_ab_* values.

### 3.4 Sensory analysis

**Figure 2:**
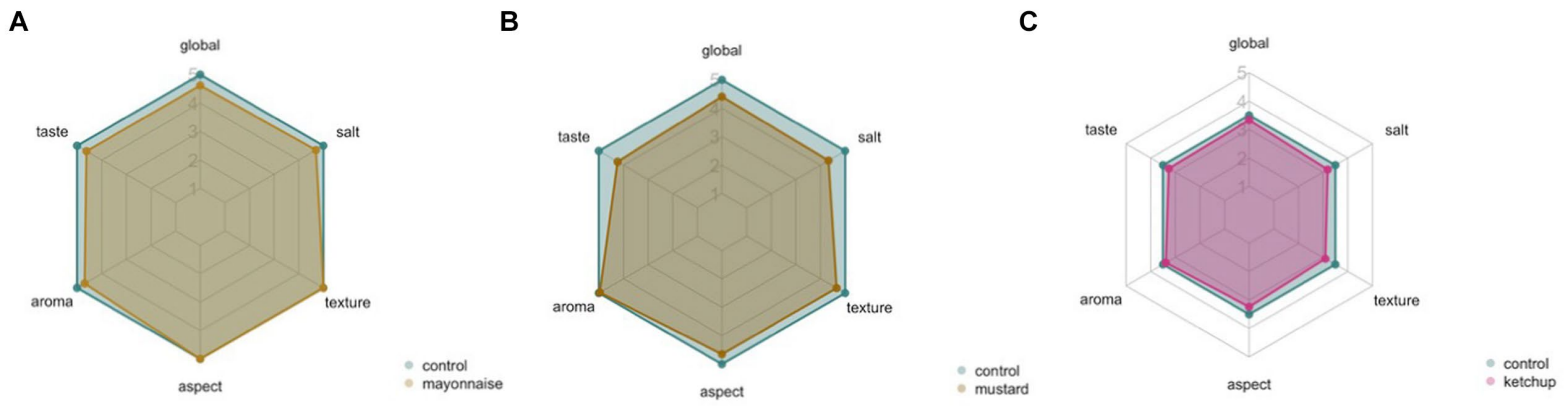
Sensory profile of mayonnaise **(A)**, mustard **(B)** and ketchup **(C)** sauces (after processing, *t* = 0). The results are expressed as mean values ± SD. A 5-point intensity scale was used, where 1 represents the worst and 5 is the best, with 5 indicating “equal to target.” The evaluation parameters included: “global evaluation,” “taste,” “smell,” “appearance,” “texture,” and “salt.”

The tasters rated the products positively overall, with an average score of around 5 on the hedonic scale of 1–9, that is, sensory analysis of all products revealed profiles similar to the control for all parameters (aroma, flavour, appearance, saltiness, texture and overall appearance) evaluated (Figures 2A–C). For mayonnaise, no significant differences were found between the formulations with ME oleoresins and the control, with this sauce being the one that most resembles the control.

While mustard presents slightly lower scores when compared to the control, mainly for flavour (*x* = 4.1 ± 1.4) and salt (*x* = 4.2 ± 1.3). Regarding ketchup sauce, the same trend was observed as for mayonnaise.

It was also found that there was no statistically significant difference between the samples.

By combining the results obtained, it was found that there was no preference for one sample over the other, so the panel members considered, based on the parameters evaluated, in the three new products, that they were not perceived by consumers, nor did they affect their acceptance.

These products are sauces used to enhance the flavour of foods in which salt is a relevant component. Several efforts have been made to reduce salt content of different foods, but no optimal solution has been found yet, and most of the studies highlight the perceived changes in taste due to the lack of salt or altered taste attributed to the substitute used (6, 9, 40). The fact that there were few differences in sensory analysis when compared to the control, highlights the potential of the formulations to reduce the salt content of each product.

## 4 Conclusion

The incorporation of microencapsulated (ME) oleoresins in mayonnaise, mustard, and ketchup formulations successfully achieved significant salt reductions, ranging from 25 to 52%, while maintaining compliance with sodium guidelines set by the EU Pledge. The potassium content in mustard and ketchup was sufficient to support health claims related to maintaining normal blood pressure, highlighting the nutritional benefits of these reformulated sauces. Additionally, the reformulated sauces demonstrated lower chloride content, better aligning with dietary reference values and offering potential health benefits by reducing the risk of hypertension and cardiovascular diseases.

The study also revealed that the ME ingredients had minimal impact on the essential physical–chemical parameters of the sauces, such as pH, lipid oxidation, and viscosity, indicating that the basic integrity and texture of the products were preserved. However, some selective changes were observed, particularly in the consistency and chloride content of the sauces. Inulin, used as a carrier agent for the ME ingredients, played a crucial role in maintaining the rheological properties of the mayonnaise, further supporting the viability of these formulations.

Colour analysis showed that the ME ingredients had varying effects depending on the sauce. The colour difference was minimal in mayonnaise, moderate in mustard, and more pronounced in ketchup, particularly in darker formulations. Despite these variations, the sensory analysis confirmed that the overall sensory profiles of the reformulated sauces closely resembled the control samples, with mayonnaise showing the closest similarity. Mustard displayed slightly lower sensory scores, particularly in flavour and saltiness, though these differences were modest.

Overall, this study demonstrates that ME ingredients can be effectively used in condiment reformulation to achieve significant salt reduction while maintaining sensory quality, supporting health claims, and meeting regulatory standards. These findings underscore the potential of ME formulations to offer healthier condiment options without compromising consumer satisfaction.

Future research should focus on the long-term stability, microbial safety, and broader consumer acceptance of these reformulated products, while also assessing their impact on food safety and shelf-life for wider adoption.

The market for these sauces has a very interesting volume to invest in innovation and development. Thus, the formulation of innovative sauces can bring added value to the company that launches them, by presenting a tasty and “traditional” product, with a low salt content, meeting the current expectations and consumer’s needs.

## Data Availability

The raw data supporting the conclusions of this article will be made available by the authors, without undue reservation.
